# Characteristics of malignant thyroid lesions on [^18^F] fluorodeoxyglucose (FDG)-Positron emission tomography (PET)/Computed tomography (CT)

**DOI:** 10.1016/j.ejro.2021.100373

**Published:** 2021-08-19

**Authors:** Hatem Nasr, Hussein Farghaly, Abdullah Alqarni, Seham Al-Salem, Mohamed Sayed

**Affiliations:** a Department of Oncology and Nuclear Medicine, Faculty of Medicine, Cairo University, Cairo, Egypt; b Department of Radiology, Prince Sultan Military Medical City, Riyadh, Saudi Arabia; c Department of Oncology and Nuclear Medicine, Faculty of Medicine, Assiut University, Assiut, Egypt

**Keywords:** F-18 FDG PET/CT, Thyroid incidentalomas, Thyroid malignancy, Lesional Hounsfield units (HU), Lesional SUVmax

## Abstract

**Objectives:**

To determine the imaging variables that can best differentiate malignant from benign thyroid lesions incidentally found on F-18 FDG PET/CT scans.

**Methods:**

All F-18 FDG PET/CT studies starting from 2011 to end of 2016 were reviewed for incidental thyroid lesions or metabolic abnormalities. Only patients who were found to have FNAB or histopathology were included. Patients with known thyroid malignancy were excluded. Patients were analyzed for age, sex, SUVmax, non-enhanced CT tissue density in mean Hounsfield units (HU), uptake pattern (focal or diffuse) and gland morphology (MNG or diffuse). A control group of 15 patients with normal thyroid glands were used to assess the tissue density in HU for normal thyroid tissue. Sensitivity, specificity, PPV, NPV and accuracy to detect malignancy were calculated. Pearson Chi-square test was used to compare categorical variables while unpaired T-test and one way ANOVA test were used to compare means of continuous variables. ROC analysis was used to assess the best cut off points for SUVmax and HU. Regression analysis was used to detect the independent predictors for malignant lesions.

**Results:**

Biopsy was unsatisfactory or indeterminate in 4/48 patients (8%). Only 44 patients (mean age 55.2 ± 14.7; 30 females (68 %)) with unequivocal FNAB or histopathology were included for further analysis. MNG was noted in 17/44 patients (38.6 %). Thyroid malignancy was found in 16/44 (36.4 %), benign thyroid lesions in 28/44 (63.6 %). Thyroid malignancies were 12 papillary, 1 follicular, 1 Hurthle cell neoplasm and 2 lymphoma. Benign lesions were 23 benign follicular or colloid nodules and 5 autoimmune thyroiditis. Focal FDG uptake pattern was more frequently associated with malignant lesions compared to benign lesions (75 % vs. 43 %; p = 0.039). The mean SUVmax and tissue density (HU) were both higher in malignant than benign lesions (8.8 ± 8.3 vs. 3.6 ± 1.9, p = 0.024) and (48.9 ± 12.7 vs. 32.9 ± 17.5, p = 0.003) respectively. The mean HU in the control group with normal thyroid tissue was 90 ± 7.4 significantly higher than in both the benign and malignant lesions (p < 0.001). ROC analysis revealed SUVmax cutoff of >4.7 and HU cutoff of >42 to best differentiate malignant from benign lesions. The sensitivity, specificity, PPV, NPV and accuracy to detect malignancy for SUVmax>4.7 were 68.8 %, 78.6 %, 64.8 %, 81.5 & 75.0 % (p = 0.002), for HU > 42 were 81.3.0 %, 75.0 %, 65.0 %, 87.5 & 77.3 % (p = 0.0003) and for both parameters combined were 87.5 %, 60.7 %, 56.0 %, 89.5 % and accuracy of 70.5 % (p = 0.002) respectively. Only HU > 42 and SUVmax>4.7 were independent predictors for malignancy with odd ratios 8.98 and 4.93 respectively.

**Conclusion:**

A higher tissue density (HU > 42) and SUVmax>4.7 as well as tendency for focal uptake pattern are the most significant characteristics associated with malignant thyroid lesions occasionally detected on PET/CT.

## 1 Introduction

The widespread use of whole body Fluorine-18 (^18^F)-Fluorodeoxyglucose (FDG)-Positron Emission Tomography (PET)/Computed Tomography (CT) in the work-up of oncological patients has led to the discovery of unexpected incidental lesions including thyroid incidentalomas (TI). Although several previous studies described TI on FDG PET/CT in patients with no known history of thyroid pathology, the incidence and clinical significance of FDG-avid TI on FDG- PET/CT remain a debatable topic [1].

The incidence of 18 F-FDG–avid TI has been reported to range from 0.2–8.9%, among patients who underwent F-18 FDG PET or F-18 FDG PET/CT for evaluation of a non-thyroidal malignancy, with a great statistical heterogeneity between studies and geographical areas [2].

As thyroid glucose uptake can be nonspecific, the prevalence of malignancies amongst thyroid incidentalomas is still uncertain. The rate of malignancy has been reported to range between 10.3 and 80.0 % of FDG TI [[3], [4], [5], [6], [7], [8], [9], [10], [11]]. In F-18 FDG PET/CT, TI may appear as a focal FDG uptake or as diffuse thyroid uptake. Several studies have reported that focal thyroid uptake on FDG PET is associated with a significant risk of malignancy [5,[12], [13], [14]]. On the other hand, diffuse thyroid uptake on FDG PET/CT has been considered more often benign [4,5,15,16]. Malignant cells tend to have higher glucose metabolism and thus may have positive F-18 FDG PET/CT scans. Although they tend to have higher maximum standardized uptake values (SUVmax) than benign nodules, the definitive cut-off SUVmax for the prediction of a malignant thyroid nodule has not yet been defined, and accurate characterization of these unexpected FDG avid thyroid findings remains a challenge [17,18].

To avoid patient anxiety, additional costs, and potential risks associated with further investigation and surgical management of 18 F-FDG–avid TI, an additional diagnostic tool besides SUVmax is required [19]. The use of Hounsfield unit (HU) values on the low-dose CT (LDCT) of F-18 FDG PET/CT to discriminate between benign and malignant TI has been suggested. However, only few studies have addressed the utility of HU values for prediction of malignant TI [14,19,20].

The purpose of this study was to determine the imaging variables that can best differentiate malignant from benign thyroid lesions incidentally found on F-18 FDG PET/CT scans.

## 2 Materials and methods

All procedures followed were in accordance with the ethical standards of the responsible committee on human experimentation (institutional and national) and with the Helsinki Declaration of 1964 and its later amendments.

### 2.1 Patients

Following approval by the institutional ethics committee, with waiving the requirement for obtaining informed consent for this retrospective analysis; all F-18 FDG PET/CT studies starting from January 2011 to end of 2016 were retrospectively reviewed for incidental thyroid lesions or metabolic abnormalities. Only patients who were found to have fine-needle aspiration biopsy (FNAB) or histopathology were included. Patients with known thyroid malignancy were excluded. A control group of 15 patients with normal thyroid glands were used to assess the tissue density in HU for normal thyroid tissue.

### 2.2 F-FDG PET/CT image acquisition and reconstruction

All patients underwent whole body 18 F-FDG PET/CT scan around 60 min post tracer injection. All imaging and data acquisition were performed using a Gemini TF 16 slice PET/CT scanner with patient port of 70 cm (Philips Medical Systems). The patients were instructed to fast except for water for 4−6 hours, and had blood glucose levels *<*180 mg immediately prior to radiotracer administration according to our local guidelines. The 18 F-FDG dose administered IV was approximately 5.18 MBq/kg (0.14 mCi/kg) of 18 F-FDG with a maximum dose of 444 MBq (12 mCi). During the subsequent 40−60 min following injection (uptake phase), patients were advised to remain seated or recumbent calmly in a quiet room, covered with a blanket to avoid uptake of the radiotracer at physiological sites as brown fat, which can result in image artifacts. During image acquisition patients were instructed to avoid motion and were allowed to breath normally without specific instructions. Emission data were acquired for 11–14 bed positions. Emission scans were acquired in a three-dimensional (3D) mode at 1 min/bed position and increased up to 2 or 3 min/bed position in case of obese patients according to patient’s body mass index (BMI). The 3D whole body acquisition parameters consisted of a 128 × 128 matrix and an 18 cm FOV with a 50 % overlap. An imaging field of view (FOV) from top or base of the skull to mid-thigh with the arms above the head whenever possible was used or otherwise the arms were positioned over the chest. Low dose CT scans were used for attenuation correction purposes and to help in anatomic localization of 18 F-FDG uptake. The CT scan was performed as a single sweep adjusted to 120–140 kV, 50–100 mA (based on BMI), 0.5 s per CT rotation, pitch -1.675:1, slice thickness of 5 mm and 512 × 512 matrix. CT acquisition was performed before the emission acquisition. CT data were used for image fusion and the generation of the CT transmission map. No intravenous contrast was used.

### 2.3 Image analysis and semi-quantitative evaluation

Visual and semi-quantitative analysis of 18 F-FDG PET/CT scans were performed. All 18 F-FDG PET/CT scans in our study population were reviewed by two nuclear medicine physicians. Any suspicious 18 F-FDG avid thyroid lesion in 18 F-FDG PET/CT was evaluated and correlated with histopathology result, recorded and tabulated. In this study, a suspicious thyroid lesion was defined as either an increased thyroid F-18 FDG uptake on PET images or a focal thyroid lesion on CT images regardless of F-18 FDG uptake. A focal F-18 FDG uptake was defined as a localized uptake occupying less than a single entire thyroid lobe while an uptake involving at least a whole thyroid lobe was analyzed in this study under the category of diffuse uptake. All included patients had at least one thyroid lesion/nodule on CT and no patients with morphologically normal thyroid glands were included.

Images were analyzed for SUVmax, uptake pattern (focal or diffuse) and non-enhanced CT tissue density in mean Hounsfield units (HU) [measured using a circular ROI at the center of the suspicious F-18 FDG avid thyroid lesion, and whenever possible corresponding to the site of highest SUVmax and avoiding areas of gross calcification].

### 2.4 Statistical analysis

All data were analyzed using SPSS software (SPSS 20.0) and MedCalc version 11 software (MedCalc, Mariakerke, Belgium). Data are presented as mean and standard deviation (SD) (mean ± SD). The best cut off values for SUVmax and HU to differentiate benign from malignant thyroid lesions were set based on ROC analysis. Data analyzed included in addition; age, gender and 18 F-FDG uptake pattern (focal versus diffuse). Suspicious thyroid lesions were correlated with biopsy, and only histopathology is accepted as a proof of malignancy. Pearson Chi-square test was used to compare categorical variables while unpaired T-test and one way ANOVA test were used to compare means of continuous variables. The sensitivity, specificity, negative predictive value (NPV), positive predictive value (PPV), and accuracy of focal uptake pattern, SUVmax, and HU in differentiation between malignant and benign thyroid lesions were calculated. Logistic regression analysis was performed for statistically significant variables to identify the most powerful independent predictors for malignant thyroid lesions. Forward stepwise method was used with entry significance level set to p *<*0.05 and removal significance level set to p*>*0.10. A P value *<*0.05 was considered statistically significant.

## 3 Results

**Fig. 1:**
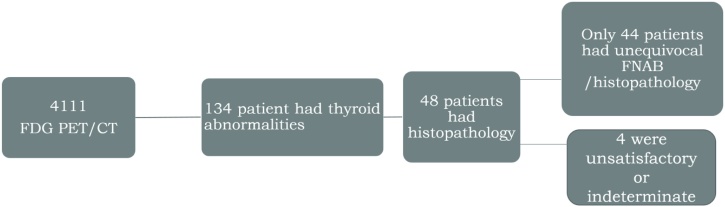
Flow chart of the included study population.

Out of 4111 18 F-FDG PET/CT studies, thyroid abnormalities were detected in 134 patients. Histopathology could be retrieved for 48/134 patients (36 %). Biopsy was unsatisfactory or indeterminate in 4/48 patients (8%). Only 44 patients [mean age 55.2 ± 14.7; 30 females (68 %)] with unequivocal FNAB or histopathology were included for further analysis (Fig. 1).

**Fig. 2:**
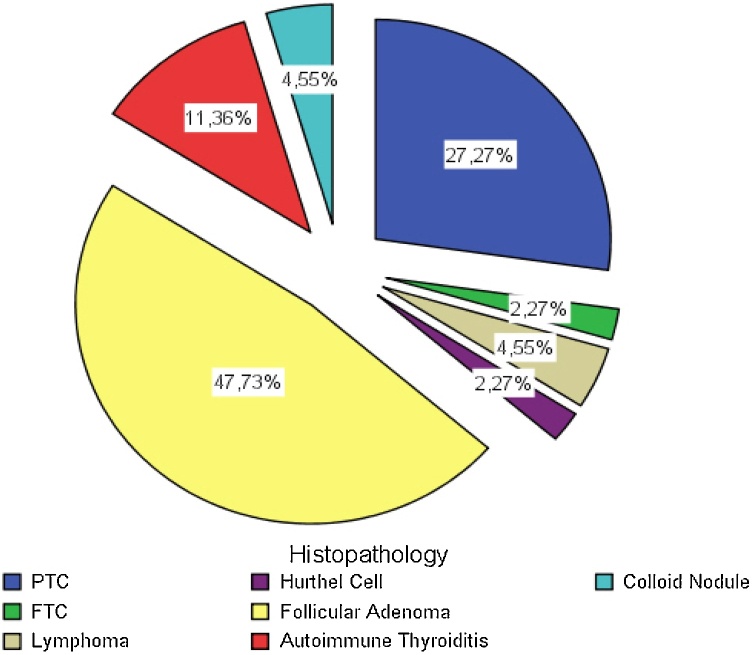
Histopathology results for the entire study population. PTC = Papillary Thyroid Carcinoma; FTC = Follicular Thyroid Carcinoma.

Multinodularity was noted in 17/44 patients (38.6 %). Thyroid malignancy was found in 16/44 (36.4 %), benign thyroid lesions in 28/44 (63.6 %). Thyroid malignancies were 12 papillary, 1 follicular, 1 Hurthel cell neoplasm and 2 lymphoma, while benign lesions were 23 benign follicular or colloid nodules and 5 autoimmune thyroiditis (Fig. 2).

Focal FDG uptake pattern was more frequently associated with malignant lesions 12/16 (75 %) compared to benign lesions 12/28 (43 %) (p = 0.039). Diffuse uptake pattern involving at least one entire thyroid lobe was noted in 20/44 patients (45.5 %) while focal uptake pattern was noted in 24/44 patients (54.5 %). All patients had at least one underlying thyroid lesion/nodule on the non-enhanced CT portion of the study, with multinodularity noted in 17 patients. In the current study none of the patients including those with diffuse thyroid uptake, had normal thyroid morphology on CT. Among 20 patients with diffuse thyroid uptake 4 had underlying malignant lesions.

**Fig. 3:**
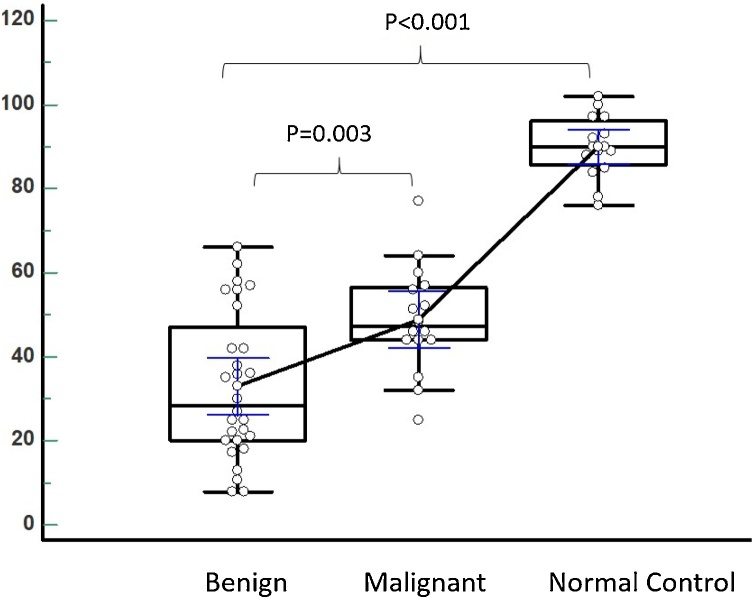
Box and Whisker graph for comparison of tissue density in mean HU between benign lesions, malignant lesions and controls with normal thyroid glands. All data plotted as dots with data connected at their means and error bars representing the 95 % CI.

The mean SUVmax and tissue density (HU) were both higher in malignant than benign lesions (8.8 ± 8.3 vs. 3.6 ± 1.9, p = 0.024) and (48.9 ± 12.7 vs. 32.9 ± 17.5, p = 0.003) respectively. The mean HU in the control group with normal thyroid tissue was 90 ± 7.4 significantly higher than in both benign and malignant lesions (p < 0.001) (Fig. 3) (Table 1).

**Table 1:** Comparison of clinical characteristics and PET metabolic parameters between patients with malignant thyroid and benign lesions.

	Confirmed malignant lesions (n = 16)	Benign lesions (n = 28)	p-value
Age	50.94 ± 12.68	57.43 ± 14.90	0.146
Sex			
Male	3 (6.8 %)	11 (25.0 %)	0.159
Female	13 (29.5 %)	17 (38.6 %)	
FDG uptake pattern			
Focal uptake pattern	12 (50 %)	12 (50 %)	0.039
Diffuse uptake pattern	4 (20 %)	16 (80 %)	
Mean SUVmax	8.89 ± 8.3	3.6 ± 1.9	0.024
Mean HU	48.9 ± 12.7	32.9 ± 17.5	0.003

The sensitivity, specificity, PPV, NPV and accuracy to detect malignancy based on focal uptake pattern versus diffuse pattern was 75.0 %, 57.1 %, 50.0 %, 80.0 & 63.6 % (p = 0.039).

**Fig. 4:**
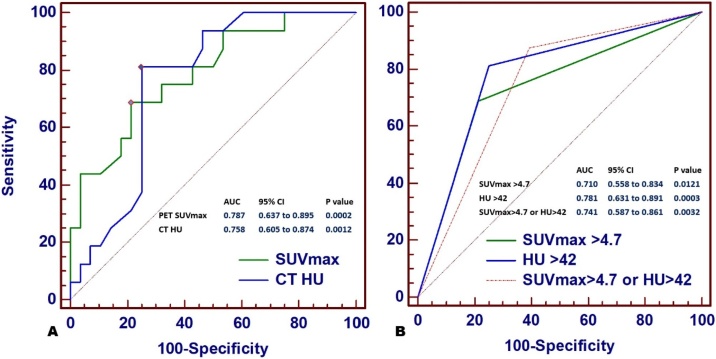
Comparison of ROC curves for SUVmax and mean HU as continuous variables (A), as well as SUVmax>4.7, HU > 42 and combined SUVmax>4.7 and HU > 42 as categorical (binary) variables (B). All curves with statistically significant AUC but comparison of AUC between different curves were not statistically significant.

ROC analysis yielded SUVmax>4.7 as an optimal cut-off to identify malignant thyroid lesions with area under the curve (AUC) of 0.787 (95 % CI 0.637 to 0.895, p = 0.0002). The cut-off for the mean HU to detect malignant thyroid lesions was 42 with AUC of 0.758 (95 % CI 0.605 to 0.874, p = 0.0012). The difference between the AUC of SUVmax>4.7 and that of mean HU value did not reach statistical significance (0.787 vs. 0.758, p = 0.772). The combined SUVmax>4.7 and HU > 42 was slightly more sensitive but less specific in differentiation between malignant and benign thyroid lesions with AUC of 0.741, (95 % CI 0.587 to 0.861, p = 0.0032) (Fig. 4).

The sensitivity, specificity, PPV, NPV and accuracy to detect malignancy for SUVmax>4.7 were 68.8 %, 78.6 %, 64.8 %, 81.5 & 75.0 % (p = 0.002), and for HU > 42 were 81.3 %, 75.0 %, 65.0 %, 87.5 & 77.3 % (p = 0.0003). The sensitivity, specificity, PPV, NPV and accuracy to detect malignancy for both parameters combined were 87.5 %, 60.7 %, 56.0 %, 89.5 % and accuracy of 70.5 % (p = 0.002) respectively (Table 2).

**Table 2:** Performance of SUVmax and HU cut-offs in distinguishing benign versus malignant thyroid lesions.

	Sens.	Spec.	PPV	NPV	Acc.	p-value
**SUVmax>4.7**	68.8 %	78.6 %	64.8 %	81.5 %	75.0 %	0.002
**HU > 42**	81.3 %	75.0 %	65 %	87.5 %	77.3 %	0.0003
**SUVmax>4.7 or HU > 42**	87.5 %	60.7 %	56 %	89.5 %	70.5 %	0.002

Regression analysis revealed that HU > 42 and SUVmax>4.7 were the only independent predictors for malignancy with odd ratios 8.98 and 4.93 respectively and overall model Chi-square of 17.8 (p < 0.001) (Table 3).

**Table 3:** Logistic regression analysis revealed HU > 42 and SUVmax >4.7 were both independent predictors for malignancy.

	Chi-square	p-value	Exp(B)	95.0 % C.I.
**HU > 42**	13.700	0.00021	8.979	1.810 - 44.530
**SUVmax >4.7**	4.132	0.045	4.928	1.038 - 23.410
**Total Model**	17.832	0.00013		

**Case 1:** Diffuse Thyroid Uptake

**Figure:**
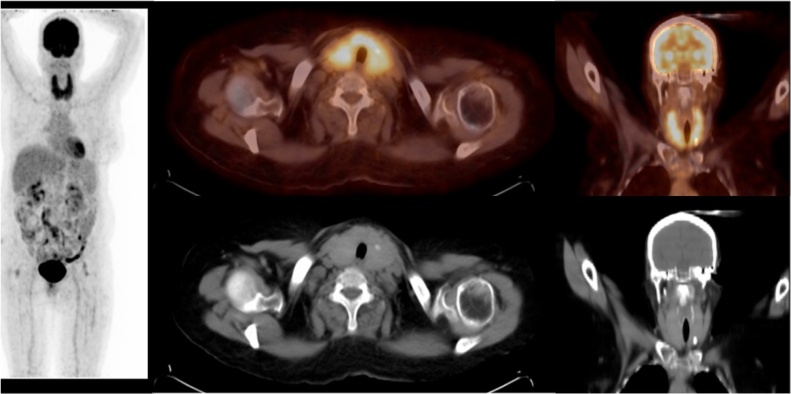

62 years old female with DM, HTN and cervical lymphadenopathy. F-18 FDG PET/CT showed diffusely enhanced FDG thyroid uptake, slightly heterogeneous CT morphology with diffusely reduced thyroid gland density, left lobe focal calcification, SUVmax of 4.6 and CT HU of 52. FNA cytological revealed autoimmune thyroiditis.

**Case 2:** Focal Thyroid Uptake

**Figure:**
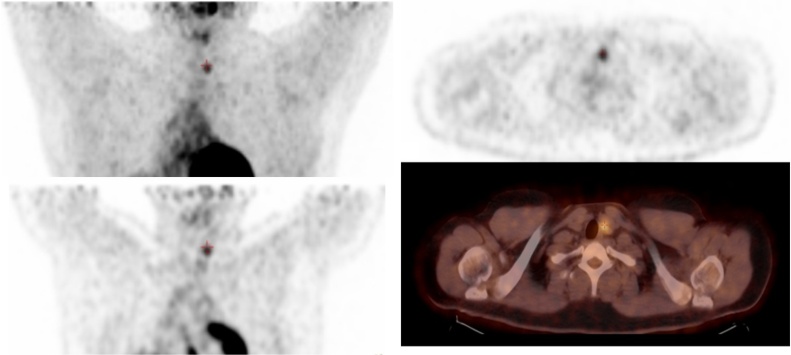

20 years old male, a case of atypical carcinoid, status post-surgical resection of right middle and lower lung lobes. F-18 FDG PET/CT showed a hypermetabolic left thyroid lobe nodule with SUVmax of 4.2 and CT HU of 32. FNA cytology revealed a benign follicular nodule.

**Case 3:** Focal Thyroid Uptake

**Figure:**
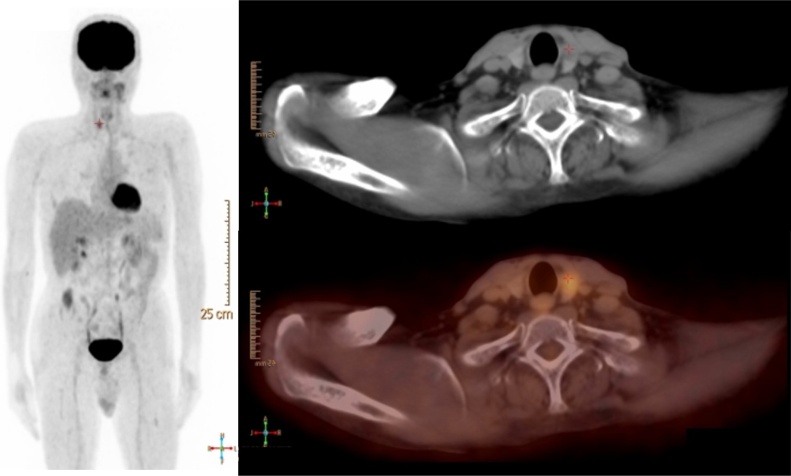

52 years old male with Wegner’s granulomatosis and left buccal squamous cell carcinoma. F-18 FDG PET/CT showed a hypermetabolic hypodense right thyroid lobe nodule with SUVmax of 5.7 and CT HU of 64. FNA cytology revealed papillary thyroid cancer.

## 4 Discussion

18-F FDG PET/CT is a molecular imaging modality provides functional and morphological information that reflect the biological behavior and provides information on the anatomical structure of the lesion [21]. As a consequence of the widespread use of FDG-PET/CT in clinical practice, incidental thyroid FDG uptake has been increasingly identified, which substantiates clarification of their clinical significance [18].

In current study, thyroid abnormalities were detected in 3.26 % (134/4111) of patients. The pooled incidence of 18 F-FDG–avid TI was 2.46 % and ranged from 0.2–8.9%, among 147,505 patients, as reported by *Bertagna et al.,* in a meta-analysis of a large number of studies published until April 2012 about the diagnosis and clinical significance of F-18-FDG-PET or F-18 FDG-PET/CT TI, with a great statistical heterogeneity between studies and geographical areas. The pooled incidence of TI found in Asia, North America, and other studies was 3.00 %, 1.83 % and 2.05 %, respectively [2]. *Nayan and colleagues (2014)* [3] conducted another systematic review and meta- analysis that included 31 studies and a total of 197,296 patients and reported FDG-avid TI in 1.85 % of subjects (range between 1.2 % and 4.3 %.) 2,4,6. The inconsistent results in the medical literature may be due to differences in glucose metabolism among detected lesions and/or differences in lesion detectability as a consequence of tumor size.

The relative clinical impact of an incidental, asymptomatic thyroid cancer in the context of active non-thyroidal malignancy is unknown but critically important information to guide the interpretation and management of this finding [22]. In current study, thyroid malignancy was found in 16/44 (36.4 %) of TI. *Bertagna et al.,* reported malignant lesions in 34.6 % of TI detected by F-18 FDG PET or PET/CT, and the malignancy ratio of TI was 32.5 %, 37 %, and 38 % in Asia, North America, and other studies, respectively [2]. *Nayan et al.* reported malignant lesions in 37 % of TI [3].

We found that malignant lesions including papillary, follicular, Hurthle cell neoplasm and lymphoma in 27.3 %, 2.3 %, 2.3 %, and 4.5 % of the cases, respectively, and no focal hypermetabolic lesion in the thyroid representing distant spread from a primary lesion was identified in the current study. *Nayan et al.* reported papillary, follicular, Hurthle cell, medullary, and anaplastic thyroid cancer in 29 %, 2.10 %, 0.67 %, 0.60 %, 0.15 %, respectively, and the pooled proportion of metastatic disease from a primary other than thyroid was 2%. The pooled proportion of non-thyroid malignancies such as lymphoma was 1% [3]. Consistent with our results that the most common pathology of TI was papillary thyroid cancer.

In accordance with previously published studies, we found that focal FDG uptake pattern was more frequently associated with malignant lesions. Among 24 patients with focal uptake pattern 12 (50 %) had malignant lesions compared to only 4 malignant out of 20 patients with diffuse uptake pattern (20 %), (p = 0.039) [23].

Malignant cells have accelerated metabolism and tend to have high glucose requirements and thus may have increased FDG uptake, as a glucose analogue labelled with F-18. The up-regulation of specific glucose transporters may represent a key mechanism by which cancer cells may achieve increased glucose uptake to support the high rate of glycolysis [24]. It has also been suggested that the SUVmax is influenced by different grades of inflammation, blood flow, and the size of the malignant lesions [7].

Normal thyroid tissue generally demonstrates low FDG uptake. A defining characteristic of thyroid cancer cells is their strong ability to take up enormous amounts of glucose compared to normal thyroid tissue for promotion of cell growth and survival. Tumor cells enhance glucose uptake across the plasma membrane via induction of a family of facilitative glucose transporter proteins (GLUTs), which is classified regarding their tissue-specific distribution and different affinities for glucose and remarkably different transport capacities. In most cases thyroid cancer cells frequently show overexpression of especially the hypoxia-responsive GLUT1 and GLUT3 proteins. Malignant cells characteristically have a reduced ability to use oxidative metabolism, and instead aerobic glycolysis increased rapidly and oxidative phosphorylation remained stable. Increased glycolysis is the main source of energy supply in cancer cells but, due to the lower energy yield of the glycolytic pathway, malignant cells show an increased rate of glucose transport across the plasma membrane to compensate the acquired energy [[25], [26], [27], [28], [29]].

It has been reported that diffuse F-18 FDG uptake is usually due to benign processes, such as thyroiditis or more rarely Graves’ disease [30,31]. To the best of our knowledge, in the literature to date, only two cases of diffuse FDG uptake in TI on PET were related to malignancy; one case harboured a papillary carcinoma associated with Hashimoto’s thyroiditis [30], and the other case was thyroid metastasis from lung cancer [32].

In the current study population the relatively high incidence of malignancy (20 %) among patients with diffuse FDG uptake pattern could be partially related to our definition of diffuse uptake, which in our study was considered to include any increased FDG uptake involving at least a single entire lobe in comparison to other studies in which diffuse is usually defined as FDG uptake involving the entire thyroid gland. Moreover, in the current study, despite diffuse FDG uptake pattern, none of our patients had normal thyroid morphology on CT portion of the study.

Hashimoto’s thyroiditis is caused by an immune response to thyroid antigens. The mechanism of FDG uptake in Hashimoto’s thyroiditis is not clearly known [30]. Increased glucose transporters have been proposed as one of the reasons why malignant cells have increased FDG accumulation. However, this phenomenon is not tumor specific. Inflammatory cells also increase the expression of glucose transporters when they are activated [[33], [34], [35], [36]]. The result of inflammatory reactions may affect thyroid FDG uptake in Hashimoto’s thyroiditis. Lymphocytes within the thyroid glands are reported to be the source of TPO antibodies as well [37]. Karantanis et al. [15] noted no correlation between the TPO antibody titers and maximum SUV (SUVmax) in their cases; therefore, additional mechanism, for example cell apoptosis and active formation of fibrosis, may also contribute to an increase of FDG uptake [16]. The enhancement of cell death is considered to be caused by lymphocytic infiltration, targeting follicular epithelia owing to autoimmune phenomena [38]. Enhanced cell death in chronic thyroiditis might include cell necrosis as well as apoptotic cell death [38,39]. Apoptosis may play an important role in cancer development, and malignant transformation of Hashimoto’s thyroiditis by increased cell death and cell proliferation caused by chronic lymphocyte infiltration. Therefore, the risk of cancer related to diffuse thyroid uptake as observed by FDG PET must be recognized [30].

Graves’ disease may demonstrate increased blood flow, enhanced glucose metabolism, and autoimmune antibody inducing inflammation, which are all factors of increased FDG uptake in the thyroid [40]. In Chen’s report [37], 14 of 22 (63.6 %) subjects with Graves’ disease had visual uptake intensity greater than or equal to liver uptake.

Focal uptake can be due to either a benign or a malignant nodule [41]. Indeed, many benign lesions could present a high FDG avidity such as Hurthle cell adenomas [2,42,43] probably because of their high number of mitochondria [44]. Other causes of very intensely F-18 FDG avid benign TI include degeneration nodules [45], follicular adenomas, and adenomatous hyperplasia [46]. Thyroiditis and pseudonodular thyroiditis may exhibit focal FDG uptake and not only diffuse TI. Thuillier et al. [42] reported that 4 of the 24 benign focal TI corresponded to a cytological aspect of thyroiditis. A recent study demonstrated that 2 of the 31 focal TI described in FDG PET/CT showed a focal aspect of Hashimoto’s thyroiditis [47].

Though, increased FDG uptake may be seen in both benign and malignant thyroid conditions, there is difference in the expression of hypoxia-related GLUT1 and GLUT3 between benign and malignant neoplasms, as well as non-neoplastic thyroid lesions. The differences in GLUT1 and GLUT3 expression levels are associated with the histological type of thyroid carcinomas as well [26,48].

According to our results, the mean SUVmax was higher in malignant than benign lesions (8.8 ± 8.3 vs. 3.6 ± 1.9, p = 0.024), and ROC analysis revealed SUVmax cutoff of >4.7 to best differentiate malignant from benign thyroid lesions. The overall accuracy of SUVmax as a binary variable (SUVmax > 4.7) to detect malignancy was 75% compared to 63.64% for focal FDG uptake pattern as the criterion of malignancy. The specificity improved from 57.1% to 78.6%, PPV from 50.0% to 64.8%, and NPV from 80.0% to 81.5%, however with some drop in sensitivity from 75.0% to 68.8%.

Many SUVmax cutoff thresholds have been proposed to distinguish benign from malignant TI, but no safe cutoff has been identified. Pérez et al. [49] reported SUVmax 4.2 as the optimal threshold to discriminate malignancy with an area under receiver-operating characteristic curve of 0.76 (95 % confidence interval, 0.60–0.90). Haydardedeoglu et al. [19] reported cut-off value for SUVmax to be 5.55.

Some of previous publications reported a statistically significant difference between SUVmax of benign lesions and the value of malignant ones [4,14,[50], [51], [52], [53]], whereas other studies did not [5,18,32,46]. This issue is still debated, and no definitive conclusion could be drawn. In fact, despite many studies had suggested that the level of SUV is predictive of thyroid malignancy, there is often overlap in SUV values between benign and malignant thyroid incidentalomas. For example, F-18 FDG avidity of certain benign thyroid lesions like Hurthle cell adenomas are found to be responsible for F-18 FDG uptake, associated with high SUV [43,44,54]. Moreover, the studies varied a lot regarding the method for calculation of the SUV value in terms of the level of fasting serum glucose, length of fasting period before the examination, volume and activity of injected F-18 FDG, time from the radiotracer administration, and PET technology. As a consequence, there are patient, technique, and procedure variations, and no reliable comparison of SUV could be done. On the basis of all these results and considerations, no SUVmax cutoff can be considered safe to discriminate benign from malignant TIs [2].

Some authors proposed the evaluation of other parameters in addition to SUVmax of the thyroid incidentalomas and the pattern of F-18 FDG uptake (focal versus diffuse), such as the target/background, the target/blood-pool, and the target/liver ratios [55].

Dual time point PET imaging has been proposed as alternative method to overcome the low specificity of SUVmax in the differentiation of benign from malignant lesions, including thyroid incidentalomas [56]. Recently, texture analysis of medical images provided numerous quantitative and semi-quantitative parameters capturing the inhomogeneity of the tissues, better characterize lesions, as well as provide some prognostic information about the aggressiveness of disease [57]**.** Kim and Chang 2015 [58] evaluated some parameters, including a feature named “heterogeneity factor”, derived from the histogram of intensities of uptake within the lesion, in patients with a thyroid nodule. Sollini et al. (2017), reported that F-18 FDG PET/CT texture analysis seems to be a promising approach to stratify the patients with thyroid incidentalomas identified on PET scans, with respect to the risk of the diagnosis of a malignant thyroid nodule and thus, could refine the selection of the patients to be referred for cytology. However, all these approaches were not validated and, they are not generally accepted [59].

Few studies have addressed the utilization of HU values on LDCT of F-18 FDG PET/CT for prediction of malignant TI [14,19,20,60,61]. In the current study, both benign and malignant lesions are relatively hypodense compared to normal thyroid gland density of control group. However, the mean tissue density (HU) was significantly higher in malignant than benign lesions (48.9 ± 12.7 vs. 32.9 ± 17.5, p = 0.003). The mean HU in the control group with normal thyroid tissue was 90 ± 7.4 significantly higher than in both the benign and malignant lesions (p < 0.001) (Table 1). ROC analysis revealed HU cutoff of >42 to best differentiate malignant from benign thyroid lesions. The sensitivity, specificity, PPV, NPV and overall accuracy to detect malignancy for HU > 42 were 81.3 %, 75.0 %, 65.0 %, 87.5 & 77.3 % (p = 0.0003), and all were higher than those of SUVmax >4.7 except for specificity. Our findings were in agreement with those recently reported by *Lee et al.* who found TI in 2.8 % of 1941 patients that underwent LDCT for lung cancer screening, and reported malignancy in 12.7 % of those TI. The positive and negative predictive values of chest LDCT for the detection of incidental malignant thyroid nodules were 26.9 % and 73.4 %, respectively. A mean attenuation value of 55 HU or more (p = 0.036) and the presence of dense calcifications (p = 0.048) considered to be predictive factors of malignancy on LDCT. Sex, age, location of the nodule, longest diameter of the lesion, AP/T (anteroposterior/transverse dimension) ratio, margins, density, presence of punctate calcifications, and thyroid enlargement had no significant predictive value in discriminating benign and malignant nodules. On multivariate analyses, a mean attenuation value above 55 was the only statistically significant feature (p = 0.048) [20]. In accordance with that, Choi et al., [14] as well reported that most of the malignant TI (88.9 %; 16/18) had low attenuation on CT and all focal thyroid lesions with a very low attenuation (HU < 25) on CT, were benign. They reported that very low attenuation, or no discernible thyroid nodule on CT favored benign thyroid lesions regardless of the SUV value.

On the other hand, Haydardedeoglu et al., [19] and Sayman et al. [60], reported no significant difference between mean HU value of benign and malignant TI. The HU measurement seems to have no additional value for the differentiation of malignant and benign thyroid nodules detected on PET/CT scans [19,60].

In contrast, Kim et al., [61] reported that the mean HU ratios of the thyroid nodule compared to contralateral thyroid lobe (T/B_HU_) on non-contrast CT component of F-18 FDG PET/CT was significantly lower in malignant TI than that of benign nodules. The AUC of T/B_HU_ was higher than that of SUVmax value ratios of TI compared to liver (T/B_SUV_) (0.941 vs. 0.689, p < 0.0001). The sensitivity, specificity, and accuracy of T/B_HU_ were significantly higher than those of T/B_SUV_ (100 % vs. 77.8 %, p = 0.0313; 80.0 % vs. 60.0 %, p = 0.0433 and 86.6 % vs. 65.9 %, p = 0.0041, respectively). The risk of malignancy was much higher (71.1 %) in TI with a T/B_HU_ cutoff value ≤0.68, whereas it was 0% in TI with a T/B_HU_ of >0.68. They concluded that T/B_HU_ is a simple and effective parameter to stratify the risk of malignancy in TI found on PET/CT and it could be of value in TI with non-diagnostic or undetermined cytologies on FNAB [61].

The sensitivity, specificity, PPV, NPV and accuracy to detect malignancy for both SUVmax>4.7 and HU > 42 parameters combined were 87.5 %, 60.7 %, 56.0 %, 89.5 % and accuracy of 70.5 % (p = 0.002) respectively.

Regression analysis revealed that only HU > 42 and SUVmax>4.7 were independent predictors for malignancy with odd ratios 8.98 and 4.93 respectively. The anatomical information of the low-dose CT provides an additional diagnostic tool besides SUVmax [19]. This help to appropriately categorize most TI as benign or of unlikely clinical significance and reduce patient anxiety, additional costs, and potential risks associated with further investigation and surgical management of F-18 FDG–avid TI [19]. Nevertheless, metastatic thyroid cancer is rare, accounting for less than 1% of thyroid malignancy in most clinical series [19,20], and the overall outcome of most oncology patients with F-18 FDG–avid TI is likely determined by the underlying malignancy, given the excellent prognosis associated with thyroid cancer especially that papillary thyroid carcinoma is the most frequently detected pathology in malignant TI.

## 5 Limitations

First, the retrospective design of the study may render selection bias unavoidable. Second, this is a single-center study with a limited number of subjects predominantly oncology patients performing PET/CT for staging or follow up. Further more, only patients with unequivocal FNAB or histopathology were included in the study which may had induced a sort of selection bias. Future prospective multi-center studies in a larger group of patients, may be considered to validate our findings, better differentiate malignant from benign incidental thyroid lesions and to avoid unnecessary more invasive procedures.

## 6 Conclusion

Higher tissue density (HU >42) and SUVmax >4.7 as well as tendency for focal F-18 FDG uptake pattern are variables highly predictive of malignancy in thyroid lesions incedentally detected on F-18 FDG PET/CT.

## Ethical statement

1) This material is the authors' own original work, which has not been previously published elsewhere.
2) The paper is not currently being considered for publication elsewhere.
3) The paper reflects the authors' own research and analysis in a truthful and complete manner.
4) The paper properly credits the meaningful contributions of co-authors and co-researchers.
5) The results are appropriately placed in the context of prior and existing research.
6) All sources used are properly disclosed (correct citation). Literally copying of text must be indicated as such by using quotation marks and giving proper reference.
7) All authors have been personally and actively involved in substantial work leading to the paper, and will take public responsibility for its content.

## Funding statement

This research did not receive any specific grant from funding agencies in the public, commercial, or not-for-profit sectors.

## CRediT authorship contribution statement

**Hatem Nasr:** Conceptualization, Methodology, Image analysis, Formal analysis, writing (Original draft preparation). **Hussein Farghaly:** Writing, Reviewing and Editing. **Abdullah Alqarni:** Supervision, Project administration, Reviewing and Editing. **Seham Al-Salem:** Data collection and management, Abstract preparation. **Mohamed Sayed, MD:** Writing, Reviewing, Editing and validation.

## Declaration of Competing Interest

The authors report no declarations of interest.
